# Symbiosis is woman: the pioneering role of women scientists

**DOI:** 10.3389/fpls.2026.1730069

**Published:** 2026-03-26

**Authors:** Paola Bonfante

**Affiliations:** Department of Life Sciences and Systems Biology, University of Turin, Turin, Italy

**Keywords:** symbiosis, gender, women scientists, plant symbioses, cooperation, competition, Lynn Margulis

## Abstract

Symbiosis is a concept that has been profoundly shaped by the contributions of women scientists, among whom Lynn Margulis stands as a leading figure. Through her vision and determination, Margulis drew scholarly attention to symbiosis, offering a transformative interpretation of the evolution of living organisms. This mini-review highlights the essential contributions of women researchers to the study of symbioses, tracing the shift from naturalistic and descriptive studies to molecular, genetic, and omics-based approaches.

## Introduction

The term symbiosis provides an ideal starting point for understanding the impact of women researchers across disciplines ranging from natural sciences and plant biology to microbiology, biotechnology, and environmental studies. The word derives from the Greek *συμβίωσις* (symbiósis), composed of *σύν* (with, together) and *βιόω* (to live), and thus conveys the notion of living together: a relationship requiring a shared physical environment in which two individuals interact.

The term was introduced into the scientific field by Heinrich Anton de Bary, a German mycologist of the nineteenth century, and has since been widely adopted in the natural sciences, later extending metaphorically into the social sciences (from economics to psychology). In its strict biological sense, symbiosis refers to interspecific interactions—relationships between individuals of different species—whereas, when applied to humans, it often concerns intraspecific interactions. This distinction marks an important conceptual transition.

Another key difference lies in the moral connotation of the term. In scientific usage, symbiosis is descriptive and value-neutral: it refers to a stable association between organisms whose outcome may be positive, negative, or neutral. From an evolutionary perspective, however, relationships that confer reciprocal benefit are more likely to persist and become widespread, enhancing the survival and the success of both the partners involved. Hamilton’ law (1964) demonstrates how mechanisms like kin selection (helping relatives who share their genes) are evolutionary strategies that promote the spread of beneficial relationships.

In contrast, humans tend to attribute ethical or moral value to natural phenomena; thus, symbiosis is often perceived as a cooperative and mutually beneficial association. Nature, however, does not conform to human moral categories—it is neither good nor bad.

To avoid such semantic ambiguity, scientists frequently specify mutualistic symbiosis when both partners benefit and display synergistic behavior—for instance, the association between the hermit crab and the sea anemone and pathogenic interactions when one partner’s health is adversely affected, as in many plant–fungal pathogenic relationships.

Despite early interest in the natural sciences, the concept of symbiosis remained somewhat marginal during much of the twentieth century and did not strongly influence the major theoretical debates in biology. Charles Darwin, for instance, was unaware of the microbial world and, during the very years when lichens were being described, was articulating the foundational principles of biology through his On Origin of Species (1859). Although Darwin devoted considerable attention to the relationships between plants and pollinators—interactions that arguably contributed to the evolutionary success of angiosperms—these associations do not constitute symbioses in the strict sense. Symbiosis implies a stable, intimate relationship that generates a new biological entity, a true novelty in evolution, as Margulis would later define it. On the other hand, plants and pollinators surely offer an excellent example of co-evolution where both the interacting organisms evolve specific adaptations (Simón-Porcar et al., 2024).

By considering the dynamic context of research in symbiosis, the aim of this minireview is to provide a short list of women scientists who have had a broad impact in this specific area. On one hand, the selection mirrors my personal/subjective interests for some aspects (symbiosis vs endosymbiosis; plant-bacterial and plant-fungal interactions); on the other it would identify a few examples of women who may offer role models for younger generations thanks to their scientific achievements sometimes reached notwithstanding an oppositive environment.

## Lynn Margulis: a flag-bearer of symbiosis

Born in 1938 in Chicago, Lynn Margulis pursued her studies in biology, culminating in a PhD at the University of California, Berkeley, in the mid-1960s. At a time when neo-Darwinism dominated biological thought and nuclear DNA was considered the exclusive repository of heredity, Margulis advanced a bold alternative: cellular evolution was driven by symbiosis between microorganisms.

Her thinking drew inspiration from Konstantin Mereschkovsky, a Russian botanist and lichenologist who, in the early twentieth century, proposed the Theory of Symbiogenesis. In his papers, Mereschkovsky hypothesized that chloroplasts were derived from once free-living cyanobacteria. Building on these largely overlooked ideas, Margulis—drawing on her expertise in microbiology—proposed that both mitochondria and plastids originated as independent, free-living bacteria. After entering a proto-eukaryotic host cell, these endosymbionts gradually lost their autonomy and became organelles, retaining only a small portion of their original DNA as a molecular signature of their prokaryotic ancestry.

The detection of DNA within chloroplasts of *Chlamydomonas* (Ris and Plaut, 1962) provided critical support for Margulis’ thinking and inspired her to develop her Serial Endosymbiosis Theory (SET). Her manuscript, “On the Origin of Mitosing Cells”, was initially rejected by several journals before being published in Journal of Theoretical Biology (Sagan, 1967). At that time, she used the surname of her first husband, the astrophysicist Carl Sagan, while she later published under her second husband’s name. Margulis also proposed a third symbiotic event involving the (9 + 2) basal bodies of flagella, hypothesizing that they too derived from once free-living prokaryotes. Although this specific idea was not supported by experimental evidence, her broader theory was gradually validated by accumulating molecular and genomic data, particularly through the sequencing of chloroplast and mitochondrial genomes. Her synthesis was later consolidated in the influential book on Symbiosis in Cell Evolution (Margulis, 1981), which played a key role in establishing symbiosis as a central principle in evolutionary biology.

Subsequent research has refined and extended Margulis’s vision. The transfer of genes from organelles to the nucleus has been extensively documented: Martin et al. (2002) estimated that approximately 56% of eukaryotic genes have bacterial signatures, whereas 44% display archaeal–eukaryotic affinities. These proportions are even higher in plants, reflecting their cyanobacterial endosymbiotic heritage. More recently, comparative genomics has clarified the identity of potential ancestors. Vosseberg et al. (2024) summarized current understanding of eukaryogenesis, suggesting that eukaryotic cells evolved between 1.8 and 2.7 billion years ago from archaeal ancestors (the Asgard archaea) through symbioses with bacterial partners—first with an alphaproteobacterial progenitor of mitochondria and later with a cyanobacterial progenitor of plastids. The evolution from the alphaproteobacterium to the mitochondrion required the development of the inner membrane invaginations, the cristae. Munoz-Gomez et al. (2015) demonstrated the presence of the Mic60 in the closest mitochondrion bacterial relatives. Since Mic60 is a core protein of the MICOS complex that helps shape the inner mitochondrial membrane and cristae junctions, the data suggest that the machinery responsible for synthesizing key mitochondrial phospholipids – which cooperate with cristae-shaping proteins - could have evolved from a bacterial ancestor (Venkatraman et al., 2025). However, as discussed in many reviews (Booth and Ford Doolittle, 2015) several questions, including the origin of the nucleus, remain unresolved.

Margulis’s visionary thinking established a conceptual framework that continues to inspire contemporary biology. The search for the last eukaryotic common ancestor (LECA) remains fundamental to understanding life’s complexity, while the study of endosymbiosis has opened new research directions, such as the investigation of endobacteria inhabiting insects and fungi (Bonfante, 2024). As a personal note, I would like to add that I was fortunate to meet Lynn in many meetings devoted to symbiosis, but the first time was a memorable one, when she attended a symposium organized in Torino in 1987 (Scannerini et al, 1988) and gave a lecture on Symbiosis and Evolution.

## Nancy Moran: a modern view of endosymbiosis bridging bacteria, insects and plants

In December 2010, Tokyo hosted a remarkable event that symbolized the scientific recognition of symbiosis as a central theme in biology. A group of researchers working on different symbiotic systems gathered to meet Emperor Akihito of Japan, himself a marine biologist and promoter of the prestigious International Prize for Biology. That year’s award, devoted to the *Biology of Symbiosis*, was presented to Nancy Moran, Professor at the Department of Ecology and Evolutionary Biology, Yale University, and member of the United States National Academy of Sciences. Moran was honored for her outstanding contributions to the study of the mutualistic relationship between the pea aphid and its intracellular symbiont *Buchnera aphidicola*.

*Buchnera* resides in specialized host cells (bacteriocytes) within the insect’s reproductive organs and is transmitted vertically from mother to offspring. Comparative genomics revealed that its genome is drastically reduced relative to that of free-living bacteria, confining it to an obligate biotrophic lifestyle dependent on its host (Moran et al, 2008). Yet, the association is finely balanced. Genomic analyses of both partners demonstrated striking metabolic complementarity: *Buchnera* receives sugars from the aphid while providing essential amino acids that are absent from the insect’s own biosynthetic pathways. Such complementarity has been confirmed in related systems—for example, in the glassy-winged sharpshooter and cicadas, one symbiont provides eight of the ten essential amino acids required by the host, while a second symbiont supplies the remaining two, each possessing distinct but complementary metabolic capabilities (McCutcheon et al., 2009).

These findings illustrate how the evolution of intimate and long-term symbioses can become irreversible through the development of metabolic codependence. Moran’s pioneering program integrated classical evolutionary ecology, molecular phylogenetics, and comparative genomics to demonstrate that vertically transmitted microbial symbionts undergo extreme genome reduction, accumulation of deleterious mutations, and biased nucleotide composition, all consequences of reduced effective population size and relaxed selection. Her laboratory defined the general principles of endosymbiont genome evolution: accelerated genetic drift, gene loss for free-living functions, and co-evolutionary adaptation with the host, leading to significant ecological and evolutionary outcomes. Moran’s studies also emphasized that insect-bacterial relationships often involve a third participant, the plant, which serves as both habitat and resource for the insect host. In this tri-partite framework, the plant’s physiology and chemistry influence symbiont–host interactions and evolutionary trajectories.

The 2010 ceremony in Japan also highlighted the contributions of other eminent women in symbiosis and marine environment (McFall-Ngai et al., 2013). Nicole Dubilier, Director of the Max Planck Institute for Marine Microbiology, presented her work on marine invertebrate–bacteria associations, encompassing biodiversity, ecology, and evolution. Her research on deep-sea mussels revealed the functional diversity that enables multiple symbiont strains to coexist within a single host, adapting to chemically variable environments. Margaret McFall-Ngai illustrated her studies on the mutualistic interaction between the Hawaiian bobtail squid and its bioluminescent bacterial partner *Vibrio fischeri*.

Together, these scientists exemplify how female researchers have defined the modern landscape of symbiosis biology across distinct ecological realms—from insects and plants to the deep sea and marine cephalopods. But, the symbiotic world is a small world indeed. Not only I met Nicole and Margareth in Tokyo, but I already discussed with Margareth when she attended -as a very young researcher- the 1987 Torino meeting.

## Rosemarie Honegger: lichens as a paradigm of symbiosis

Until the nineteenth century, lichens were regarded as independent taxonomic entities, classified using Linnaean binomials. In 1867, Simon Schwendener revolutionized this view by recognizing lichens as dual organisms, resulting from the association between a fungus and a photosynthetic partner (an alga or cyanobacterium). Consequently, each component follows the taxonomic rules of its respective group: the fungal partner (the *mycobiont*) typically belongs to the Ascomycota, more rarely the Basidiomycota, while the photosynthetic partner (the *photobiont*) belongs to the Chlorophyta or, in some cases, to cyanobacteria such as *Nostoc*.

The partnership leads to a true metamorphosis: the two organisms produce a new, self-sufficient structure—the *thallus*—which may be leafy, crustose, or fruticose and bears little resemblance to the free-living partners. Microscopically, the fungus forms a pseudotissue through interwoven hyphae that define upper and lower cortical layers, while algal or cyanobacterial cells are embedded between the hyphae. The photobiont performs photosynthesis, supplying carbohydrates that allow the fungus to survive in nutrient-poor environments. When the photosynthetic partner is a cyanobacterium, nitrogen fixation also occurs in specialized cells (heterocysts), providing additional resources.

Lichens thus represent a self-sustaining ecological unit, embodying Margulis’s concept of symbiosis as a generator of biological novelty. The association enables carbon fixation, nitrogen assimilation, mineral acquisition, and protection from environmental stress, allowing lichens to colonize extreme habitats. They are classic pioneer species, thriving on bare rocks, in tundra environments, and under freezing temperatures. Their poikilohydric nature permits survival through almost complete desiccation: when dry, metabolic activity declines to basal levels, resuming rapidly upon rehydration.

Lichenized fungi also secrete distinctive secondary metabolites, including lichen acids such as lecanoric and cetraric acid, which weather rocks and release mineral elements essential for growth. Despite this resilience, lichens are sensitive to atmospheric pollution. During the Industrial Revolution in England, they were observed to disappear from urban and industrial areas coated with soot and sulphur. In the latter half of the twentieth century, lichens became key bioindicators of air quality: visible symptoms of pollution include thallus discoloration, necrotic spots, and chlorophyll oxidation leading to reduced photosynthetic efficiency.

Lichens have attracted numerous women scientists who shaped the field. Margalith Galun (1927–2012), Professor of Botany at Tel Aviv University, founded the academic journal *Symbiosis* in 1985 and organized several landmark meetings, including the first International Symbiosis Congress (Jerusalem, 1991) and the International Mycological Congress (Jerusalem, 1998). Galun’s research focused on lichen development and the creation of a comprehensive lichen collection from across Israel.

In the late twentieth century, Rosemarie Honegger, Emeritus Professor at the University of Zurich, became a leading figure in lichenology. Using transmission electron microscopy, she investigated the functional morphology of lichens, considering structural analysis essential to understanding the physiological signals exchanged between mycobiont and photobiont. Her micrographs revealed a spectrum of mycobiont–photobiont interactions, ranging from wall-to-wall contact to complex intracellular haustorial penetration. From these observations, Honegger inferred the routes of water and metabolite exchange, as well as the transfer of photosynthates from the algal partner to the fungus. A central focus of her work was the study of hydrophobic molecules at the partner interface. Among her most influential papers is one on hydrophobins from the lichen-forming basidiomycete *Dictyonema glabratum* (Trembley et al, 2002). Hydrophobins are small (~100 amino acids), cysteine-rich proteins that self-assemble into amphipathic monolayers at hydrophilic–hydrophobic interfaces, altering surface wettability. These properties make hydrophobins key molecules for understanding how lichens tolerate extreme desiccation, colonize diverse substrates, and contribute to biogeochemical cycling.

Honegger’s work combined cytology, anatomy, ecology, and palaeobotany to elucidate how lichens evolved and persist in extreme habitats. Her integrative reviews synthesized the ontogeny and functional morphology of lichens, proposing that the fungal partner controls thallus architecture, photobiont distribution, and developmental transitions between filamentous and compact growth forms. She thus reframed lichens as highly regulated symbiotic systems, not merely loose associations—a perspective that continues to influence symbiosis research today.

## Éva Kondorosi: flying inside a symbiosis that helps feed the world

Nitrogen is essential for life: it is required for the growth, development, and reproduction of all living beings, since it is present in nucleic acids, amino acids, proteins, chlorophyll, and ATP molecules. In plants, which represent the first step of the food chain, nitrogen is taken up from the soil mainly as ammonium or nitrate. The latter is the plant’s preferred form, but it is often not available in sufficient quantities to guarantee optimal growth, particularly for crops. For these reasons, nitrogen fertilizers form the basis of modern intensive agriculture—an approach that carries significant environmental and energetic costs.

However, molecular nitrogen (N_2_) makes up about 78% of the atmosphere, even though it is not directly available to most living organisms, with the exception of certain microbes known as nitrogen-fixing bacteria. These bacteria, active both in the oceans and in many soils, possess the enzyme nitrogenase, which enables them to reduce atmospheric N_2_ to ammonia under ambient temperature and pressure. Many of these microorganisms live in association with the roots of some plants (mainly legumes), inducing the formation of specialized organs called *nodules*, where they fix nitrogen and release ammonia to their host plants—acting as powerful natural fertilizers.

The biology of nitrogen-fixing nodules represents one of the most fascinating chapters in plant science, offering a beautiful example of evolutionary innovation. Bacteria and plants exchange signaling molecules that are reciprocally recognized, triggering nodule morphogenesis. The colonization process is tightly regulated by a complex gene-signaling network and by the nodule’s precise anatomical organization. Since nitrogenase is highly sensitive to oxygen, nodules develop specialized cell layers that reduce internal oxygen concentration to levels compatible with enzyme activity.

Since the 1980s, a strong scientific community has been working to decipher the molecular and cellular mechanisms underlying this remarkable symbiosis. Among the many outstanding contributors, Sharon R. Long and Ann Hirsch helped define the molecular dialogue in rhizobial symbiosis, while more recently Simona Radutoiu was instrumental in identifying plant LysM receptor kinases that are critical for the recognition of rhizobial Nod-factors as well as two residues which reprogram immunity receptors for the symbiosis (Tsitsikli et al, 2025). However, one of the most relevant pioneers in molecular genetics of legume–rhizobium symbiosis has been Éva Kondorosi: together with Adam Kondorosi she has achieved exceptional results, playing a central role in advancing the molecular genetics and cell biology of the *Rhizobium–legume* nitrogen-fixing symbiosis. Kondorosi’s research elucidated the signaling exchanges that initiate nodulation—specifically how plant flavonoids and bacterial Nod factors trigger nodule development—and revealed how the host plant controls bacterial differentiation within the nodules (Kondorosi et al, 2013).

Her group characterized families of cysteine-rich nodule-specific peptides (known as NCR peptides) that the host plant uses to reprogram rhizobia into terminally differentiated, nitrogen-fixing bacteroids—a discovery with broad implications for our understanding of symbiosis, cell-cycle regulation, and even antimicrobial peptide biology.

Éva Kondorosi’s pioneering contributions have been recognized by major awards (such as the Balzan Prize) and leadership roles within European science policy and advisory bodies. And yes, she, too, was present in Tokyo in 2010! For all these reasons, Éva Kondorosi stands as a shining example of a woman in science, combining rigorous research with a deep awareness of the societal and policy dimensions of scientific work.

## Maria J. Harrison and the many mycorrhizal ladies

Involving more than 90% of land plants and soil fungi from nearly all fungal taxa, mycorrhizae represent one of the most widespread and successful symbioses on Earth. Their success is both temporal and spatial: fossil records attest to their presence in extinct plant species dating back to the Devonian era (about 500 million years ago), and they occur in virtually all ecosystems. The biodiversity of fungal networks is now considered a form of ecological capital that must be protected.

A large and dynamic scientific community has contributed to our understanding not only of mycorrhizal ecological roles in nutrient cycling and global-change processes, but also of partner’s biology and the complex plant–fungal dialogue. A pioneering role was played by Barbara Mosse, who in the 1960s laid the foundations for understanding the “unculturability” of arbuscular mycorrhizal (AM) fungi and demonstrated their positive impact on plant growth (Bonfante, 2018).

Today, one of the most prominent researchers in this field is Toby Kiers, a brilliant and talented ecologist who has brought mycorrhizal fungi to the forefront of public and scientific attention. As the founder of SPUN (Society for the Protection of Underground Networks), she has emphasized the fundamental role of mycorrhizal networks in soil health and planetary dynamics (Van Nuland et al., 2025). Thanks to her energetic advocacy, fungi are now increasingly recognized as essential to life on Earth.

At the same time, the specific field of AM symbiosis has been profoundly shaped by several outstanding women scientists. From Sally Smith, with her classical textbook (Smith and Read, 2008), to Vivienne Gianinazzi-Pearson Manuela Giovannetti,, Maria J. Harrison, and Uta Paszkowski, and extending to a long and still incomplete list of younger generations of researchers (such as Caroline Gutjahr, Luisa Lanfranco, Leonie Luginbuehl, Yina Jiang…), many women have been at the forefront of this scientific domain.

One name is exemplary: Maria J. Harrison. She, Professor at Boyce Thompson Institute and member of the U.S. National Academy of Sciences, entered the field of mycorrhizal research in the 1990s after a background in molecular plant pathology. She laid the groundwork for much of our current understanding of AM symbiosis. Harrison provided the first experimental evidence that an AM fungus expresses a phosphate transporter (Harrison and Buuren, 1995), and from that discovery she elucidated many crucial aspects of nutrient exchange.

Among her key contributions, she identified and characterized a symbiosis-specific phosphate transporter (MtPT4) in *Medicago truncatula*, showing that it is expressed specifically in arbuscule-containing cells and localized to the periarbuscular membrane. This discovery established the molecular basis for symbiotic phosphate transfer, revealing that symbiotic Pi uptake is mechanistically distinct from direct root uptake. Interestingly, Harrison’s team also demonstrated that phosphate transport is essential for arbuscule maintenance: loss of MtPT4 leads to premature arbuscule degeneration and collapse of the symbiosis, highlighting that nutrient exchange is required not only for plant benefit but also for fungal persistence within roots.

In her widely cited reviews, Harrison has provided comprehensive syntheses of early signaling discoveries (pre- and post-contact signaling, plant signaling pathways, systemic phosphate responses) and of the complex transcriptional and cell-biological programs activated in arbusculated cells. Among her many other contributions (including the identification of genes required for symbiosis establishment), Maria Harrison has shaped the conceptual framework of the field by combining creativity with rigorous experimentation.

## Conclusions

The role played by women in the field of symbiosis—particularly in photosynthetic symbioses—is impressive, even if the selection presented here inevitably reflects a personal perspective. One might ask whether there exists a gender-related sensitivity toward the concept of symbiosis as cooperation. In line with ecofeminist thought, could it be that women show a particular interest in such relational aspects of natural life?

It is not easy to provide a definitive answer. As scientists, we know that evolution follows general principles: living beings are shaped not only by competition—the “struggle for existence” described by Darwin—but also by cooperation, as emphasized by Lynn Margulis. Biological science currently recognizes how these two forces, competition and cooperation, are intricately balanced, even within the most successful symbiotic systems on our planet.
